# The inter-chamber differences in the contractile function between left and right atrial cardiomyocytes in atrial fibrillation in rats

**DOI:** 10.3389/fcvm.2023.1203093

**Published:** 2023-08-07

**Authors:** Xenia Butova, Tatiana Myachina, Raisa Simonova, Anastasia Kochurova, Elena Mukhlynina, Galina Kopylova, Daniil Shchepkin, Anastasia Khokhlova

**Affiliations:** ^1^ Institute of Immunology and Physiology, Ural Branch of Russian Academy of Sciences, Yekaterinburg, Russian Federation; ^2^ Institute of Natural Sciences and Mathematics, Ural Federal University, Yekaterinburg, Russian Federation; ^3^ Institute of Physics and Technology, Ural Federal University, Yekaterinburg, Russian Federation

**Keywords:** atrial fibrillation, left and right atria, single cardiomyocytes, sarcomere shortening, auxotonic force, ([Ca^2+^]_*i*_) transients, actin-myosin interaction, protein phosphorylation

## Abstract

**Introduction:**

The left and right atria (LA, RA) work under different mechanical and metabolic environments that may cause an intrinsic inter-chamber diversity in structure and functional properties between atrial cardiomyocytes (CM) in norm and provoke their different responsiveness to pathological conditions. In this study, we assessed a LA vs. RA difference in CM contractility in paroxysmal atrial fibrillation (AF) and underlying mechanisms.

**Methods:**

We investigated the contractile function of single isolated CM from LA and RA using a 7-day acetylcholine (ACh)-CaCl_2_ AF model in rats. We compared auxotonic force, sarcomere length dynamics, cytosolic calcium ([Ca^2+^]*_i_*) transients, intracellular ROS and NO production in LA and RA CM, and analyzed the phosphorylation levels of contractile proteins and actin-myosin interaction using an *in vitro* motility assay.

**Results:**

AF resulted in more prominent structural and functional changes in LA myocardium, reducing sarcomere shortening amplitude, and velocity of sarcomere relengthening in mechanically non-loaded LA CM, which was associated with the increased ROS production, decreased NO production, reduced myofibrillar content, and decreased phosphorylation of cardiac myosin binding protein C and troponin I. However, in mechanically loaded CM, AF depressed the auxotonic force amplitude and kinetics in RA CM, while force characteristics were preserved in LA CM.

**Discussion:**

Thus, inter-atrial differences are increased in paroxysmal AF and affected by the mechanical load that may contribute to the maintenance and progression of AF.

## 1. Introduction

The contractile function of the atria is an important determinant of ventricular filling and cardiac output (1). Atrial fibrillation (AF) is the most common cardiac arrhythmia, which results in loss of organized atrial contraction leading to depressed cardiac pump function, blood stasis, and thrombus formation (2, 3). The different roles of the left (LA) and right (RA) atria in the initiation and maintenance of AF have been widely debated. Previous studies showed that LA is typically the location of high-frequency sources in both paroxysmal and chronic AF resulting in LA-to-RA frequency differences during AF, which are associated with inter-atrial differences in structure and K^+^ and Na^+^ channel proteins (4–8). LA fibrosis may have a greater impact on AF initiation and maintenance than RA fibrosis, which also may contribute to LA-to-RA frequency differences during AF (6, 9, 10). Cai et al. showed the chamber-specific differences in NO production in pigs subjected to AF for 7 days with a significant decrease in nitric oxide (NO)·level in LA but not in RA (11). It appears that different ionic and humoral mechanisms may cause distinct sensitivity of LA and RA to AF.

Most studies have focused on the AF-associated changes in structure and electrophysiology of the atria (12–14). Much less attention has been devoted to the studies of the mechanical function of atrial myocardium in AF. Studies of the AF effects on atrial contractility were performed mainly on RA. A decrease in contractility of RA bundles isolated from AF patients was shown (2, 15, 16). It has been demonstrated that in chronic AF, active force and the kinetics of force redevelopment were reduced in human skinned cardiomyocytes (CM) from RA appendages. These alterations were associated with post-translational changes of myofilament proteins and changing isoform composition of sarcomeric proteins (15). Experiments on myofibrils from atrial samples of patients showed a change in characteristics of tension development, the myosin and titin isoform composition, and protein phosphorylation in AF (2, 15, 16). A recent proteomic study on atrial tissues from patients with AF has demonstrated that AF is associated with marked changes in the expression of contractile proteins (17). Short-term AF also alters CM contractility. The patients with paroxysmal AF, which is characterized by brief AF episodes (18, 19) and goats with rapid atrial pacing for 7 days showed decreased atrial pump function (18, 19). Using a 7-day tachypacing AF model in dogs, Wakili et al. (20) found a depressed CM shortening and decreased cytosolic Ca^2+^ concentration ([Ca^2+^]*_i_*) transients and a change in protein phosphorylation in RA.

In this study we have put forward two hypotheses: (i) there are LA vs. RA differences in CM contractile function in paroxysmal AF, and (ii) these differences are related to the chamber-specific sarcomeric dysfunction rather than to changes in electromechanical coupling. We examined LA vs. RA differences in CM mechanical function in paroxysmal AF using an acetylcholine (ACh)-CaCl_2_ induced AF model in rats. We compared auxotonic force, sarcomere shortening, and [Ca^2+^]*_i_* transients in single CM from LA and RA in control male rats and rats with AF. To investigate the molecular mechanisms underlying the CM contractility disorder in AF, we analyzed intracellular reactive oxygen species (ROS) and NO production, the characteristics of actin-myosin interaction using an *in vitro motility* assay, and the phosphorylation levels of contractile proteins. We found that in mechanically non-loaded CM, AF reduced the sarcomere shortening amplitude and sarcomere relengthening kinetics in LA but increased end-diastolic sarcomere length and the velocity of sarcomere shortening in RA providing LA vs. RA differences. Changes in the mechanical function of LA CM were associated with the reduced NO production, increased ROS production, and decreased phosphorylation of cardiac myosin binding protein C (cMyBP-C) and troponin I (TnI). In mechanically loaded CM, AF depressed the auxotonic force amplitude and kinetics in RA but not in LA indicating an influence of mechanical load on LA vs. RA responsiveness. We conclude that in the rodent heart, inter-atrial differences in morphological and mechanical characteristics increases in AF after 7 days of paroxysms that may contribute to a progression from paroxysmal to more sustained forms of AF.

## 2. Materials and methods

### 2.1. Experimental model of ACh-CaCl_2_-induced AF

All procedures involving animal care and handling were performed according to the guidelines stated in Directive 2010/63/EU of the European Parliament and approved by the Animal Care and Use Committee of the Institute of Immunology and Physiology of RAS (protocol № 06/20 from 10 November 2020). Male Wistar rats at 9 weeks of age were obtained from the animal house of the Institute of Immunology and Physiology. They were randomly divided into the groups with ACh-CaCl_2_-induced AF and age-matched intact control rats. Rats with AF and control rats were caged separately in groups of 5–6 per cage in a room at 22–24°C under a 12:12-h light-dark cycle and with unlimited access to food (Delta Feeds LbK 120 S-19, BioPro, Novosibirsk, Russian Federation) and water. Unless otherwise noted, all chemicals and reagents were purchased from Sigma-Aldrich (Merck KGaA).

Short-term AF in rats was induced using the ACh-CaCl_2_ model (21, 22) with slight modifications. Briefly, male Wistar rats (aged 9 weeks, *m* = 250–300 g) were injected daily with AChCl (60 µg/ml) and CaCl_2_ (10 mg/ml) via the tail vein at 1.3 ml/kg for 7 days. To detect AF episodes, ECG was recorded every 7 days under brief isoflurane anesthesia (Isoflutek 1,000 mg/g, Laboratorios Karizoo S.A., Barcelona, Spain) before and after ACh-CaCl_2_ injections using a three-channel electrocardiograph (ECG300G-VET, China). AF was defined as irregular supraventricular tachycardia with no visible P waves and irregular RR intervals, with a duration ≥30 s on ECG. The first AF episodes were noticed after the first injection. Rats that satisfied these criteria were used for experiments (≈95% of animals). Three rats died during the injection procedures. Directly before the experiments, ECG was recorded in the AF group again to verify the presence of AF. All control rats were in sinus rhythm. Seven days after the first ACh-CaCl_2_ injection, the rats were deeply anesthetized with an intramuscular injection of 0.3 ml/kg tiletamine + zolazepam (Zoletil 100®, Virbac, Carros, France) and 1 ml/kg Xylazine 2% (Alfasan, Woerden, Netherlands), and euthanized by exsanguination.

### 2.2. Histological studies

The hearts were fixed in 10% formalin for 24–48 h and embedded in paraffin using the embedding system Leica EG1160 (Leica Microsystems, Wetzlar, Germany). Then, the embedded hearts were cut along the long axis into thin slides (3–5 µm) using a microtome Leica SM2000R (Leica Microsystems). To assess the atrial wall thickness and the nuclei density, the paraffin slides were stained with hematoxylin and eosin (HE) using a Leica Autostainer XL (Leica Microsystems). To analyze collagen content in the atrial myocardium, the paraffin slides were dewaxed and stained with 0.1% Picrosirius red solution [Picro Sirius Red Stain Kit (ab150681), Abcam, Cambridge, UK]. The glycogen and myofibrillar contents were assessed using the periodic acid Schiff and methylene blue staining.

The width of atrial walls and the nuclei density were determined in HE-stained tissue using light microscopy and Leica Application Suite software for a minimum of 20 representative fields from each region per heart (Leica DM 2500, Leica Microsystems, 40× and 100× magnification). For the evaluation of collagen, glycogen and myofibrillar contents, we used Morphology 5.2 software (VideoTest, Saint Petersburg, Russia), analyzing a ratio of stained areas to the total area with transmitted light (Leica DM 2500, Leica Microsystems, 40× and 100× magnification).

### 2.3. Atrial CM isolation

Single CM from LA and RA were isolated using a combined technique of Langedorff perfusion and intra-chamber injections (23, 24) with slight modifications. Briefly, animals were heparinized with 5,000 IU/kg sodium heparin (Ellara, Pokrov, Russia) before euthanasia. The heart was isolated, cannulated via the aorta to the Langendorff apparatus, and perfused at a rate of 3.0–3.5 ml/min. All solutions were oxygenated with 100% O_2_ and maintained at 35.5°C. The perfusion was started with a heparinized (10 IU/ml) physiological solution (in mM: 140.0 NaCl, 5.4 KCl, 1.2 MgSO_4_, 10.0 HEPES, 20.0 taurine, 5.0 adenosine, 11.1 D-glucose, 1.0 CaCl_2_, pH 7.35) for 5 min. The perfusion was then switched to a low-Ca^2+^-high K^+^ solution (in mM: 115.0 NaCl, 14.0 KCl, 1.2 MgSO_4_, 10.0 HEPES, 20.0 taurine, 5.0 adenosine, 11.1 D-glucose, 0.3 EGTA, 0.025 CaCl_2_, pH 7.15) for 12 min. Afteward, the heart was perfused with an EGTA-free-high K^+^ enzyme solution, containing 0.8 mg/ml collagenase II (∼305 IU/ml; Worthington, Biochemical, Lakewood, NJ, USA), 0.06 mg/ml protease XIV (∼3.5 IU/ml), and 0.025 mM CaCl_2_ (pH 7.35) for 10–15 min. During the Langedorff perfusion, atria were injected with an enzyme solution containing 1.0 mg/ml collagenase II and 0.06 mg/ml protease XIV. Then the heart was removed from the Langendorff apparatus, and atria were transferred to a Petri dish for the intra-atrial injections with an enzyme solution (0.9 mg/ml collagenase II and 0.06 mg/ml protease XIV) for ≈25 min. LA and RA were separated, and atrial tissues were cut into small pieces. CM were re-suspended with an EGTA-free-high K^+^ enzyme solution supplemented with BSA (5 mg/ml), and extracellular Ca^2+^ concentration (0.1–1.0 mM) was gradually adjusted. The yield of viable single atrial CM was ≈70% for LA and RA in both control and AF groups.

For measurements of reactive oxygen species (ROS) and NO production, CM were stored in a low-Ca^2+^ modified Tyrode solution (140.0 mM NaCl, 5.4 mM KCl, 1.0 mM MgSO_4_, 10.0 mM HEPES, 11.1 mM D-glucose, and 0.025 mM CaCl_2_, pH 7.35) to prevent spontaneous contractions of atrial CM during recordings. For measurements of sarcomere shortening, auxotonic force, and [Ca^2+^]*_i_* transients, CM suspensions were stored in a modified Tyrode solution (140.0 mM NaCl, 5.4 mM KCl, 1.0 mM MgSO_4_, 10.0 mM HEPES, 11.1 mM D-glucose, and 1.8 mM CaCl_2_, pH 7.35). Isolated single CM were kept at rest for at least 30 min before being used in experiments at room temperature (22 ± 2°C) and used within 4–6 h.

### 2.4. Measurements of ROS and NO contents in atrial CM

Intracellular ROS and NO production ([ROS]*_i_*, [NO]*_i_*) in atrial CM were measured using the superoxide indicator dihydroethidium (DHE) and diaminofluorescein-FM diacetate (DAF-FM), respectively. CM were stained with 5 µM DHE at room temperature or with 5 µM DAF-FM at 37°C for 30 min in darkness and then washed with a low-Ca^2+^ modified Tyrode solution. [ROS]*_i_* and [NO]*_i_* were recorded in resting (non-stimulated) CM within 20 min after staining using a confocal laser scanning microscopy system (LSM 710, Carl Zeiss, Jena, Germany) with a 63× oil-immersion objective (Plan-Apochromat 63×/1.40 Oil DIC M27) and Zen 2010 software. The DHE was excited optically using Ar-laser at 405 nm, and emission was collected at 410–480 nm. The DAF-FM was excited using Ar-laser at 488 nm. The intensity of emitted fluorescence was collected at 495–565 nm. The analysis of confocal 2D images of stained CM was performed using FIJI ImageJ software (National Institutes of Health, Bethesda, MD, USA). To validate DHE signal stability, we also recorded the fluorescence intensity in electrically stimulated CM over a period of 20 min (25). In the end of these experiments, H_2_O_2_ (1 mM) was applied to increase ROS production (Supplementary Material Figure S1).

### 2.5. Measurements of CM geometry and sarcomere length dynamics in single atrial CM

CM width (diameter) and CM length were measured on a picture of resting CM using the IonOptix system (IonOptix Corporation, Milton, MA, USA, 40× magnification) and processed offline using FIJI ImageJ software.

Sarcomere shortening and relengthening at steady-state conditions (after 5 min of pacing at 1 Hz) during mechanically non-loaded CM contractions were measured using the IonOptix system. Only spindle-shaped CM with well-defined sarcomere striations were examined. The average sarcomere length (SL) was calculated from the intensity profile derived on the sarcomere striation pattern in a selected narrow region on the CM surface using a fast Fourier transformation-based algorithm in Ion Wizard software (IonOptix Corporation, Milton, MA, USA). Mechanically non-loaded sarcomere shortenings were recorded at a pacing frequency of 1 Hz and 30°C.

The following parameters were analyzed: end-diastolic sarcomere length (EDSL), absolute sarcomere shortening amplitude (EDSL minus end-systolic SL), fractional sarcomere shortening amplitude normalized by EDSL (FS), maximum velocities of sarcomere shortening (*v*_short_) and relengthening (*v*_rel_), time from the start of sarcomere shortening to peak shortening (time to peak shortening, TTP_S_), time from peak shortening to 50% sarcomere relengthening (TTR_S50_).

### 2.6. Measurements of [Ca^2+^]*_i_* transient in atrial myocytes

[Ca^2+^]*_i_* transients in mechanically non-loaded CM were recorded using a LSM 710 and Zen 2010 software. For imaging of [Ca^2+^]*_i_* transients, CM were incubated with 1.7 µM Fluo-8AM (AAT Bioquest, Sunnyvale, CA, USA) and 0.1% Pluronic® F-127 (AAT Bioquest, Sunnyvale, CA, USA) in darkness for 20 min at room temperature and then washed with a modified Tyrode solution. The Fluo-8AM was excited optically using Ar-laser at 488 nm. The intensity of emitted fluorescence was collected at 493–575 nm from a selected narrow region on the cell surface (3 pixels high, 200 pixels length). The Ca^2+^ content of the sarcoplasmic reticulum (SR) was assessed as the amplitude of [Ca^2+^]*_i_* transients evoked by rapid exposure to 10 mM caffeine. CM were electrically stimulated at 1 Hz > 5 min except during caffeine application experiments. Measurements of [Ca^2+^]*_i_* transients were carried out at a pacing frequency of 1 Hz and 30°C.

The changes in fluorescence signal (Δ*F*/*F*_0_, where *F*_0_ is the initial fluorescence measured at the diastolic phase of [Ca^2+^]*_i_* transients) were calculated and used as an index of the change in [Ca^2+^]*_i_* transients using custom-made software EqapAll 6 (26). The following parameters of electrically evoked [Ca^2+^]*_i_* transients were analyzed: the amplitude of [Ca^2+^]*_i_* transients (CaT), time from the start of [Ca^2+^]*_i_* increase to peak systolic [Ca^2+^]*_i_* (time to peak [Ca^2+^]*_i_* transients, TTP_Ca_), and the time from TTP_Ca_ to 50% decay of [Ca^2+^]*_i_* transients (TTD_50_).

### 2.7. Measurements of the auxotonic force of atrial CM

Measurements of auxotonic force generated by mechanically loaded atrial CM were performed as described elsewhere (27, 28). Briefly, two pairs of carbon fibers (≈10 µm in diameter, Tsukuba Materials Information Laboratory, Japan) were attached to the top and bottom surfaces of the left and right CM ends by electrostatic forces. Carbon fiber stiffness (0.07–0.09 mN/mm) was measured using a force transducer system (Aurora Scientific, Ontario, Canada). CM shortening was recorded using the IonOptix system. The active force was calculated by multiplying carbon fiber stiffness with the CM shortening, and then it was normalized to the CM cross-sectional area. Measurements were carried out at a pacing frequency of 1 Hz at room temperature. The amplitude of normalized force amplitude, maximum velocities of force development (*v*_Fdev_) and relaxation (*v*_Frel_), time to peak force development (TTP_F_), and time from force peak to 50% relaxation (TTR_F50_) were calculated using Ion Wizard software and used for the statistical analysis.

### 2.8. *In vitro* motility assay

Cardiac myosin and native thin filaments (NTF) were extracted from LA and RA according to (29, 30), respectively. F-actin was obtained from the bovine left ventricle (31). The *in vitro* motility assay experiments were performed as described in detail previously (24, 32). Briefly, 300 µg/ml myosin in an AB buffer (in mM: 25 KCl, 25 imidazole, 4 MgCl_2_, 1 EGTA, and 20 DTT, pH 7.5), containing 500 mM KCl was loaded into the flow chamber. After 2 min, 0.5 mg/ml BSA was added for 1 min. Furthermore, 50 µg/ml of non-labeled F-actin in an AB buffer with 2 mM ATP was added for 5 min. Then TRITC-phalloidin labeled F-actin at a concentration of 10 nM (by G-actin) was added for 5 min. The sliding velocity of F-actin was measured in a final AB buffer containing 0.5 mg/ml BSA, oxygen scavenger system, 20 mM DTT, 2 mM ATP, and 0.5% methylcellulose. Measurements of the NTF velocity were performed in a final AB buffer at a saturated calcium concentration (pCa 4). The experiments were carried out at 30°C and repeated 3 times. In each experiment, 7 image sequences were recorded from different fields. In each field, the movement of 7–12 filaments was tracked for at least 10 frames. The sliding velocities of ∼100 actin filaments or NTF per experiment were measured using the GMimPro software (33).

### 2.9. Analysis of protein phosphorylation

We analyzed protein phosphorylation using a 12% SDS-PAGE with Pro-Q Diamond phosphoprotein staining (Invitrogen, Eugene, OR, USA). SYPRO Ruby (Invitrogen, Eugene, OR, USA) staining was used to estimate the total amount of protein. Protein samples and gel staining were prepared according to the manufacturer's manual. The gel was scanned on the ChemiDoc MP Imaging System (Bio-Rad, Hercules, CA, USA), and band densities were determined with Image Lab 5.2.1 software (Bio-Rad, Hercules, CA, USA). A level of protein phosphorylation was expressed as a ratio of the Pro-Q Diamond intensity to the SYPRO Ruby intensity.

### 2.10. Statistical analysis

All experimental data were collected with Excel 16 (Microsoft Corp, Redmond, WA, USA) and the respective statistical analyses were performed using the R Studio software (RStudio Team, Integrated Development for R., Boston, MA, USA). The graphs were generated in GraphPrism 8.0 software (Origin Lab, Northampton, MA, USA). Data are expressed as median and interquartile range. The compliance with the normal distribution was checked by the Shapiro-Wilk test, and then data were transformed via log or square root transformation when appropriate. Hierarchical clustering analysis with the linear mixed model (34) was performed to quantify the amount of CM clustering for each rat, and appropriate corrections to the statistical significance test were applied. For characteristics where only one value from each rat was included, a Scheirer-Ray-Hare (SRH) test was performed to analyze differences between regions and conditions, followed by Bonferroni *post hoc* test. A *p*-value of <0.05 was considered to indicate a significant difference between groups.

## 3. Results

### 3.1. AF affects the differences in histological and morphological characteristics between LA and RA

**Figure 1:**
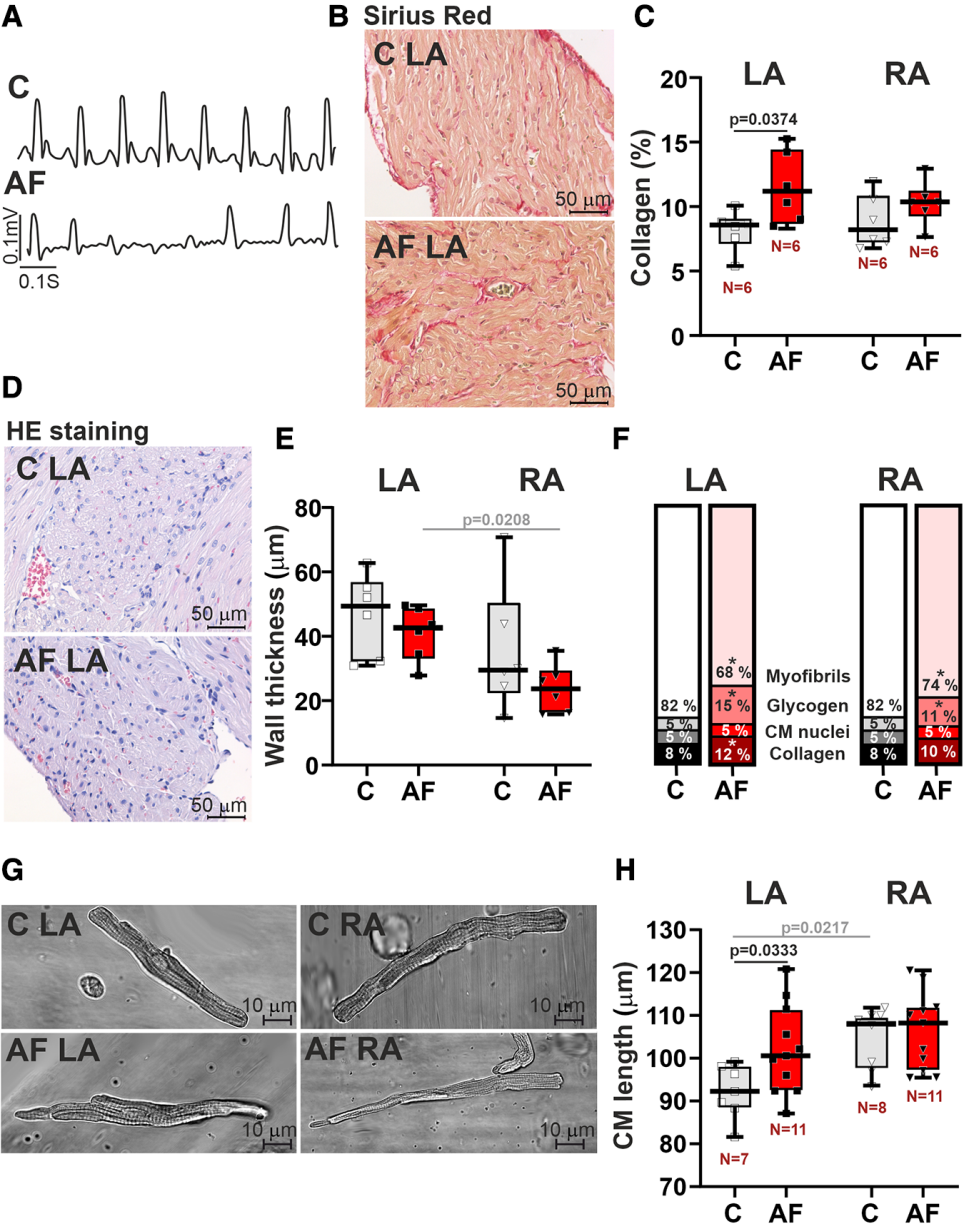
Histological and morphological characteristics of left atrial (LA) and right atrial (RA) remodeling in rats with ACh-CaCl_2_-induced AF. (**A**) Example of an atrial fibrillation (AF) episode induced by an ACh-CaCl_2_ injection where C—is the control group. (**B**) Representative Sirius Red staining of LA tissue from AF and control rats for assessment of collagen content (40× magnification). (**C**) Collagen content in LA and RA tissues using Picrosirius red staining. (**D**) HE staining of LA (40× magnification). (**E**) The thickness of LA and RA walls in control and AF rats. (**F**) Relative tissue composition in LA and RA (*indicates a significant difference between AF and control groups). (**G**) Representative images of single isolated cardiomyocytes (CM) showing elongation of LA CM in AF (40× magnification). (**H**) Length of single LA and RA CM. Data are presented in box and whisker plots, where the boxes are drawn from Q1 to Q3, horizontal lines represent median values and whiskers provide the 100% range of the values. Each dot represents a median value from one animal. The number of *N* hearts in each group is shown below the boxplot. Samples are from the same animals in (**C,E,F**). Scheirer-Ray-Hare test with Bonferroni *post hoc* test.

An example of an AF episode induced by ACh-CaCl_2_ injection is shown in Figure 1A. Representative examples of longitudinal sections of atrial muscle preparations are shown in Figures 1B,D. After 7 days of ACh-CaCl_2_ injections, there were signs of interstitial fibrosis in LA (*p* = 0.0374 for collagen content, SRH test with Bonferroni *post hoc* test, Figures 1B,C). Collagen content did not differ between LA and RA in both control and AF groups (*p* > 0.99).

In the control group, there were no differences in atrial wall thickness (*p* = 0.2188, Figure 1E), or in CM width (*p* > 0.99, Supplementary Material Figure S2) between LA and RA, while CM length was greater in RA than in LA (*p* = 0.0217, Figure 1H, SRH test). We also did not find LA vs. RA differences in glycogen (*p* > 0.71) and myofibrillar contents in the myocardial tissue (*p* > 0.99, Figure 1F).

In rats with AF, CM length in LA was increased as compared to the control animals (∼1.1 fold, *p* = 0.0333, Figures 1G,H), indicating the elongation of LA CM in AF. In both LA and RA, AF resulted in an increased content of glycogen (LA, RA: *p* = 0.0212) and a decreased myofibrillar content (LA: *p* = 0.0079; RA: *p* = 0.0326, SRH test, Figure 1F).

AF abolished the LA vs. RA difference in CM length (*p* = 0.7507, Figure 1H). Atrial wall thickness (*p* > 0.18, Figure 1E) and CM width (diameter) (*p* > 0.58, Supplementary Material Figure S2) did not differ between AF and control groups. AF resulted in the appearance of the inter-atrial difference in atrial wall thickness with LA being thicker than RA (*p* = 0.0208) and led to the difference in the myofibrillar content, which became smaller in LA than in RA (*p* = 0.0113). This together indicates that paroxysmal AF provokes profound changes in LA morphology altering the inter-atrial difference in chamber geometry.

**Figure 2:**
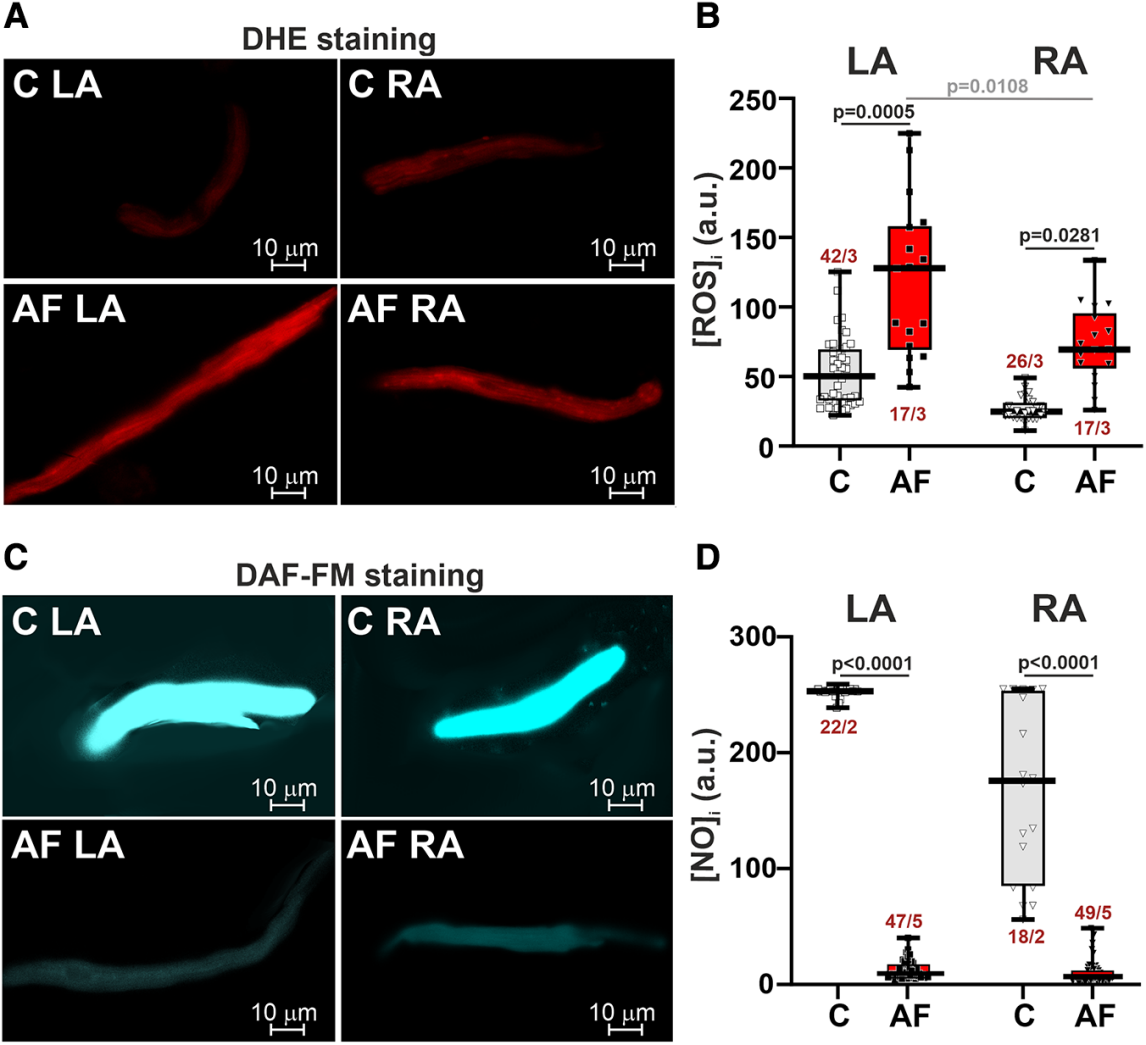
ROS and NO production in LA and RA CM in control rats and ACh-CaCl_2_-induced AF. (**A**) Representative confocal images of CM stained with the superoxide indicator dihydroethdium (DHE). (**B**) Intracellular ROS production in LA and RA CM. (**C**) Representative confocal images of CM from the control (**C**) and AF rats (AF) stained with diaminofluorescein-FM diacetate (DAF-FM). (**D**) Intracellular NO production in LA and RA CM. Data are presented in box and whisker plots, where the boxes are drawn from Q1 to Q3, horizontal lines represent median values and whiskers provide the 100% range of the values. Each dot represents an individual CM. The number of *n* CM from *N* hearts in each group is shown (5–12 CM from one rat). Statistical significance was determined by hierarchical clustering analysis with log-transformed data.

AF increased ROS production (∼2.6-fold in LA CM, *p* = 0.0005, and ∼2.8-fold in RA CM, *p* = 0.0281, Figures 2A,B) and markedly reduced NO content (∼39-fold in LA CM and ∼26-fold in RA CM, *p* < 0.0001, hierarchical clustering analysis with log-transformed data, Figures 2C,D). In the control group, ROS and NO levels were not different between LA and RA CM (*p* > 0.99). AF led to the LA vs. RA difference in ROS production with greater [ROS]*_i_* in LA CM than in RA CM (*p* = 0.0108, Figure 2A) pointing to AF-associated changes in the inter-atrial variability in ROS production.

### 3.2. Contractile dysfunction of mechanically non-loaded LA and RA CM

**Figure 3:**
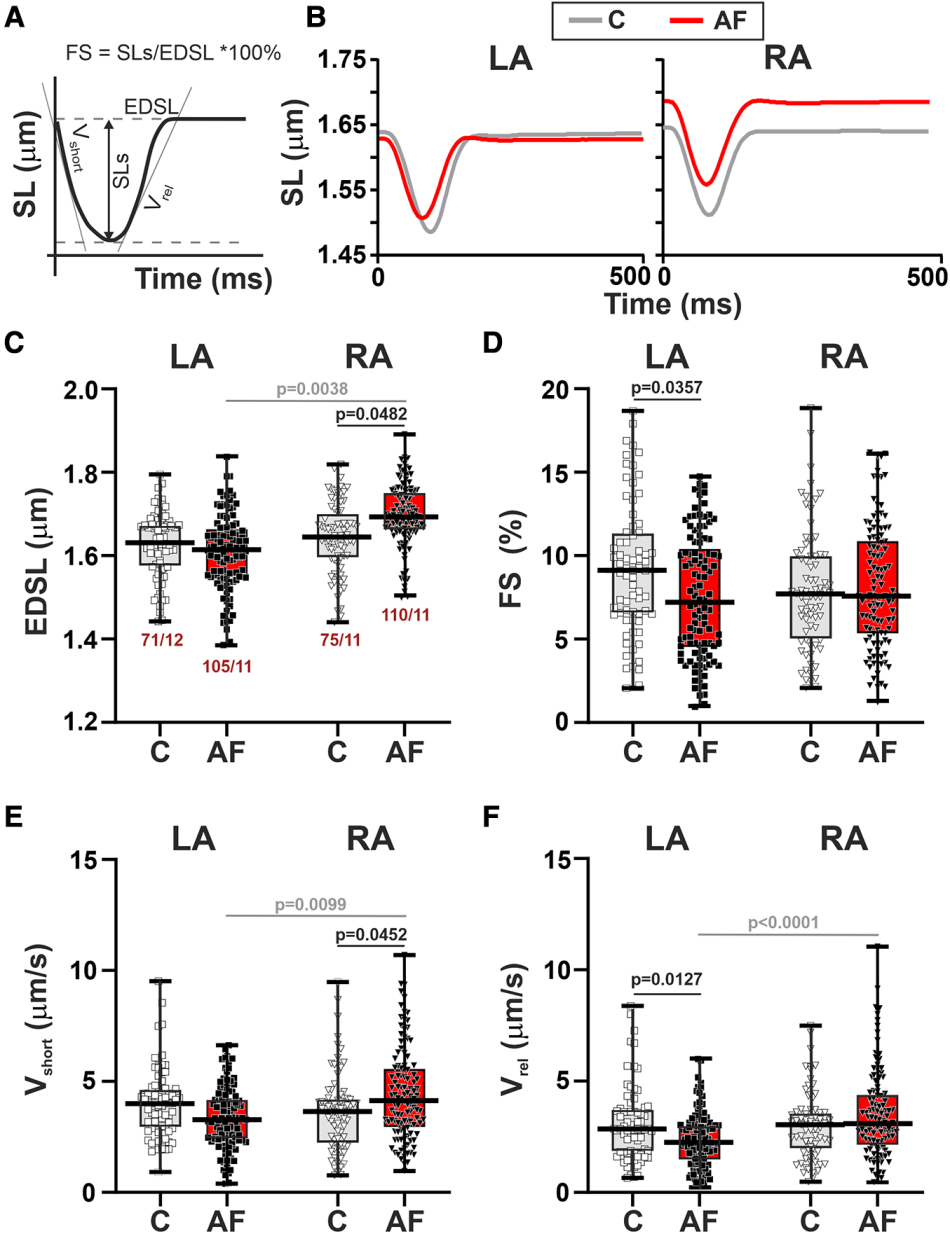
La vs. RA differences in sarcomere length (SL) dynamics in rats with ACh-CaCl_2_-induced AF. (**A**) Analyzed parameters derived from the SL change signal. (**B**) Representative recordings of the time-dependent SL changes in contracting LA and RA CM from the control rats (**C**) and AF rats (AF). (**C**) End-diastolic SL (EDSL). (**D**) Fractional sarcomere shortening amplitude (FS = sarcomere shortening amplitude/EDSL × 100%). (**E**) Maximum velocity of sarcomere shortening (*v*_short_). (**F**) Maximum velocity of sarcomere relengthening (*v*_rel_). Data are presented in box and whisker plots, where the boxes are drawn from Q1 to Q3, horizontal lines represent median values and whiskers provide the 100% range of the values. Each dot represents an individual CM. The number of *n* CM from *N* hearts in each group is shown below the first boxplot (5–14 CM from one rat). Statistical significance was determined by hierarchical clustering analysis with log or square root-transformed data.

First, we studied SL dynamics in mechanically non-loaded CM. Representative traces of mechanically non-loaded sarcomere shortening-relengthening in single LA and RA CM in the control and AF groups and analyzed parameters are shown in Figures 3A,B. We found that in mechanically non-loaded CM, AF increased EDSL in RA CM (∼1.03-fold, *p* = 0.0483, hierarchical clustering analysis, Figure 3C) without significant effects on the sarcomere shortening amplitude (*p* > 0.99, Figure 3D). In LA CM, AF provoked a decrease in both absolute (∼1.27-fold, *p* = 0.0444) and fractional (∼1.26-fold, *p* = 0.0357, Figure 3D) sarcomere shortening amplitudes compared to the control group.

Regarding the kinetics of sarcomere shortening and relengthening, AF increased *v*_short_ in RA CM (∼1.13-fold, *p* = 0.0452) and decreased *v*_rel_ (∼1.27-fold*, p* = 0.0127) in LA CM compared to the control group (hierarchical clustering analysis, Figures 3E,F). TTP_S_ (*p* > 0.88) and TTR_S50_ (*p* > 0.67) were not different between AF and control groups for both cell types (Supplementary Material Figures S3E,F).

Analyzing the inter-chamber differences in SL dynamics we observed that in control rats there were no LA vs. RA differences in either EDSL (*p* > 0.99), fractional or absolute sarcomere shortening amplitudes (*p* > 0.20), *v*_max_, *v*_rel_ (*p* > 0.99), TTP_S_ or TTR_S50_ (*p* > 0.48). AF resulted in the appearance of chamber-specific differences in EDSL with longer EDSL in RA CM than in LA CM (*p* = 0.0038, Figure 3C). AF also led to the LA vs. RA differences in *v*_short_ (*p* = 0.0099), *v*_rel_ (*p* < 0.0001), and TTR_S50_ (*p* = 0.0449) with higher *v*_short_ and *v*_rel_, and shorter TTR_S50_ in RA CM than in LA CM (Figures 3D,E, Supplementary Material Figures S3B,C).

Thus, in mechanically non-loaded CM AF led to the chamber-specific changes in SL dynamics, which induced LA-to-RA gradients in CM mechanics.

### 3.3. AF does not induce changes in [Ca^2+^]*_i_* transients

**Figure 4:**
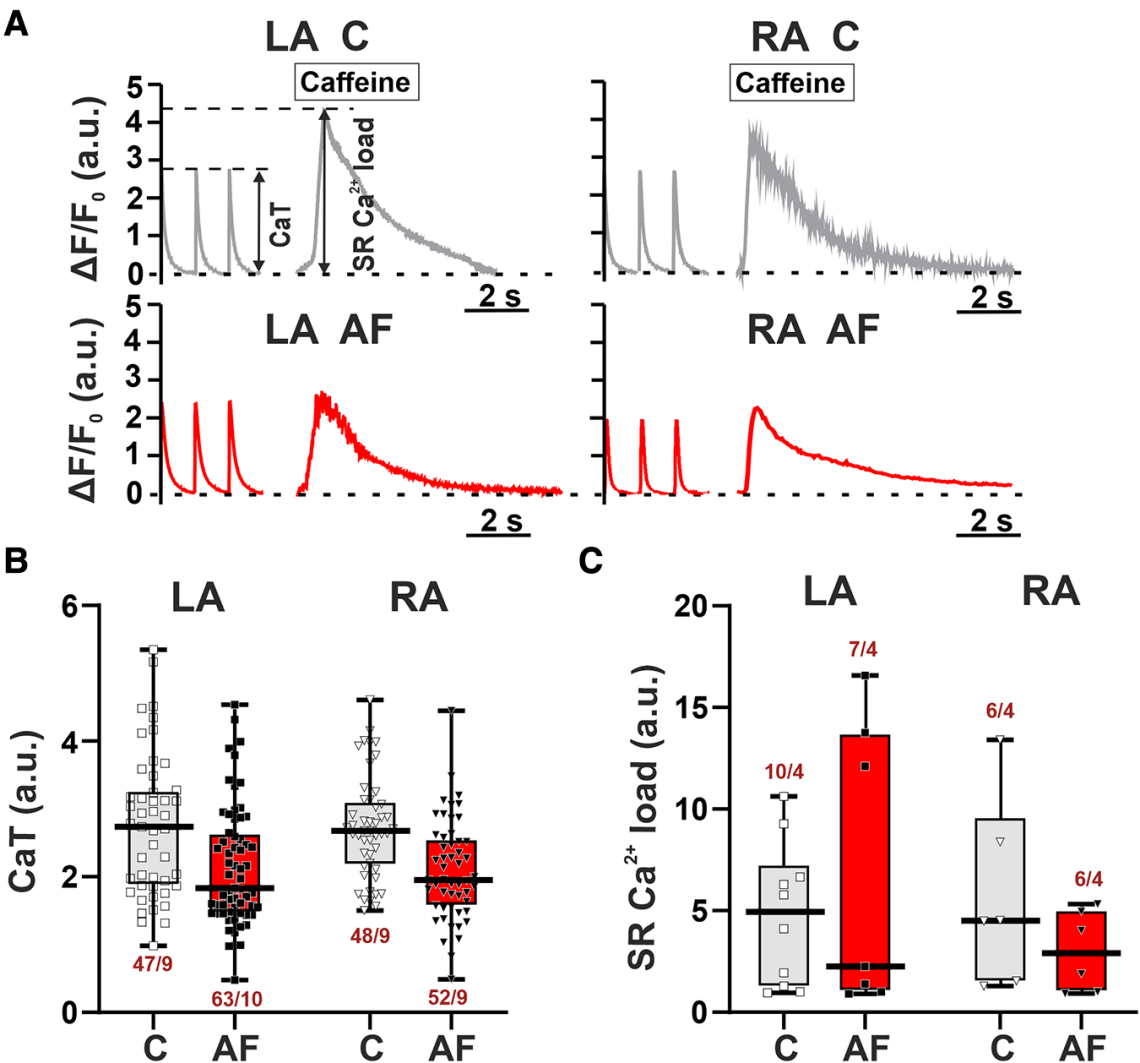
No LA vs. RA differences in Ca^2+^ handling in rats with ACh-CaCl_2_-induced AF. (**A**) Representative recordings of [Ca^2+^]*_i_* transients at a pacing frequency of 1 Hz followed by application of caffeine (10 mM) in LA and RA CM from the control rats (**C**) and AF rats (AF). F, fluorescence intensity; F_0_, fluorescence intensity at rest. (**B**) The amplitude of electrically evoked [Ca^2+^]*_i_* transients (CaT). (**C**) The sarcoplasmic reticulum (SR) Ca^2+^ load measured as the amplitude of [Ca^2+^]*_i_* transients evoked by caffeine application. Data are presented in box and whisker plots, where the boxes are drawn from Q1 to Q3, horizontal lines represent median values and whiskers provide the 100% range of the values. Each dot represents an individual CM. The number of *n* CM from *N* hearts in each group is shown below the boxplot. (4–12 CM (**B**) or 2–3 CM (**C**) from one rat). Statistical significance was determined by hierarchical clustering analysis with log-transformed data.

To study if the AF-induced alterations in sarcomere shortening in atrial CM were associated with the changes in [Ca^2+^]*_i_* dynamics, we examined the characteristics of [Ca^2+^]*_i_* transients in mechanically non-loaded CM. We also assessed SR Ca^2+^ load measured as the amplitude of [Ca^2+^]*_i_* transients evoked by caffeine application. Representative signals depicting electrically and caffeine-evoked [Ca^2+^]*_i_* transients in single LA and RA CM from control and AF groups and the analyzed parameters are shown in Figure 4A.

AF did not lead to significant changes in both the amplitude of electrically evoked [Ca^2+^]*_i_* transients (LA: *p* = 0.1211; RA: *p* = 0.3553, Figure 4B) and caffeine-evoked [Ca^2+^]*_i_* transients (LA, RA: *p* > 0.99, hierarchical clustering analysis, Figure 4C). TTP_Ca_ and TTD_50_ were not different between AF and control groups for both cell types (*p* > 0.88, Supplementary Material Figure S4). There were no LA vs. RA differences in the characteristics of [Ca^2+^]*_i_* transients in either control (*p* > 0.16) or AF groups (*p* > 0.33).

Thus, the AF-induced changes in the characteristics of contraction of LA and RA CM were not associated with the alterations in [Ca^2+^]*_i_*.

### 3.4. AF decreases the contractility of single mechanically loaded RA CM

**Figure 5:**
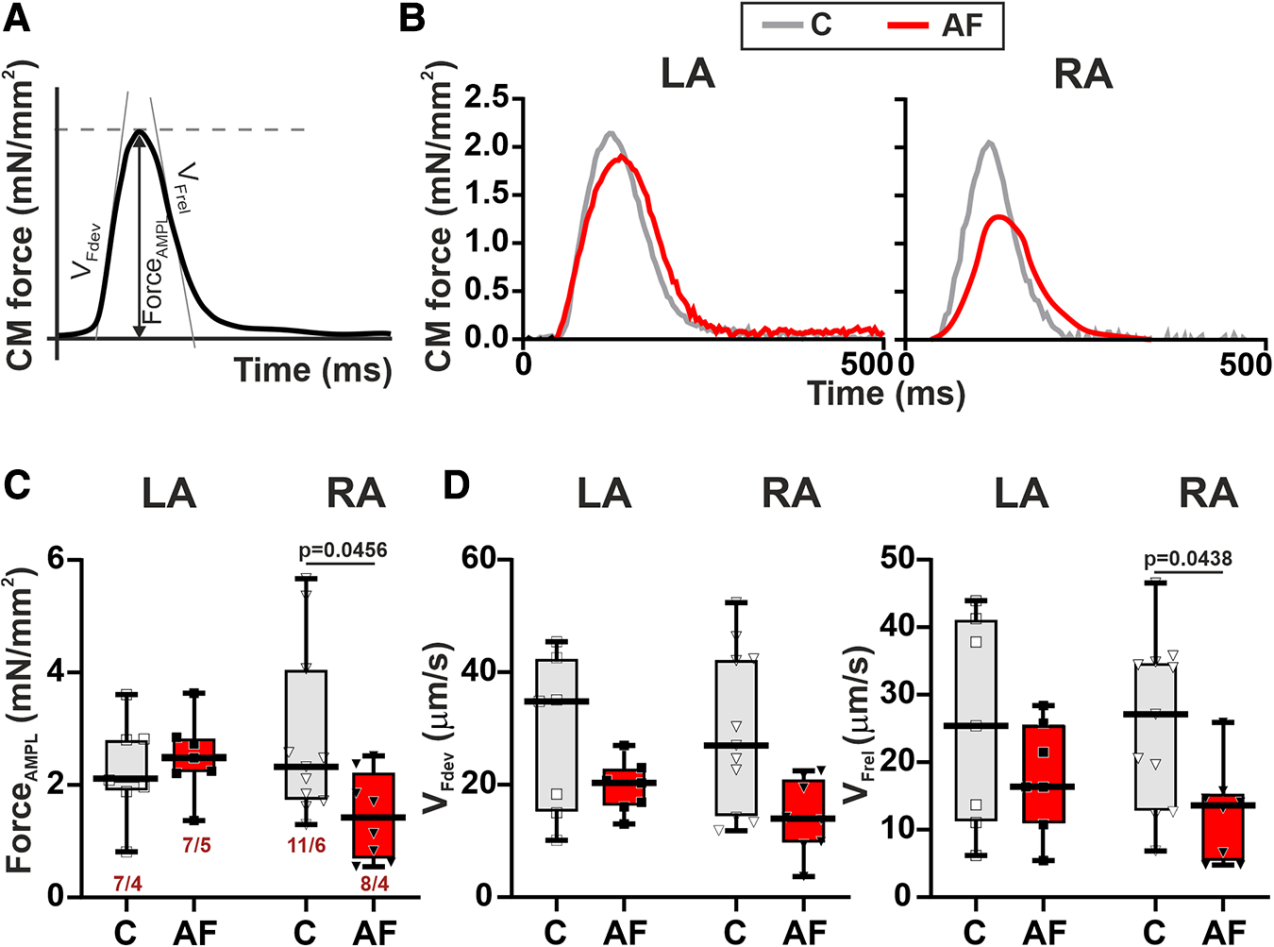
La vs. RA differences in auxotonic force characteristics in rats with ACh-CaCl_2_-induced AF. (**A**) Analyzed parameters derived from the auxotonic force signal. (**B**) Representative auxotonic force recordings in contracting LA and RA CM from the control and AF rats. (**C**) Normalized force amplitude. (**D**) Maximum velocity of force development (*v*_Fdev_). (**E**) Maximum velocity of force relaxation (*v*_Frel_). Data are presented in box and whisker plots, where the boxes are drawn from Q1 to Q3, horizontal lines represent median values and whiskers provide the 100% range of the values. Each dot represents an individual CM. The number of *n* CM from *N* hearts in each group is shown below the first boxplot (1–3 CM from one rat). Statistical significance was determined by hierarchical clustering analysis with log-transformed data.

Then we assessed the contractility (auxotonic force) of single CM mechanically loaded by carbon fibers. Representative signals of auxotonic forces and examined parameters are shown in Figures 5A,B. AF caused a ∼1.6-fold reduction in the normalized auxotonic force amplitude (*p* = 0.0456, Figure 5C) and a ∼1.9-fold decrease in *v*_Frel_ (*p* = 0.0438, Figure 5D) in RA CM but did not significantly affect these characteristics in LA CM (*p* > 0.70, hierarchical clustering analysis). AF did not influence *v*_Fdev_ (LA: *p* > 0.65, RA: *p* > 0.18, Figure 5D) or TTP_F_ and TTR_F50_ in both cell types (LA: *p* > 0.22, RA: *p* > 0.42, Supplementary Material Figures S3E,F). The force amplitude, velocity and time course parameters (*p* > 0.99) did not differ between LA and RA CM in both control (*p* > 0.99) and AF groups (*p* > 0.14).

These results demonstrate that mechanical load may affect the vulnerability of LA and RA CM to AF.

### 3.5. AF induces chamber-specific changes in sarcomeric protein phosphorylation

**Figure 6:**
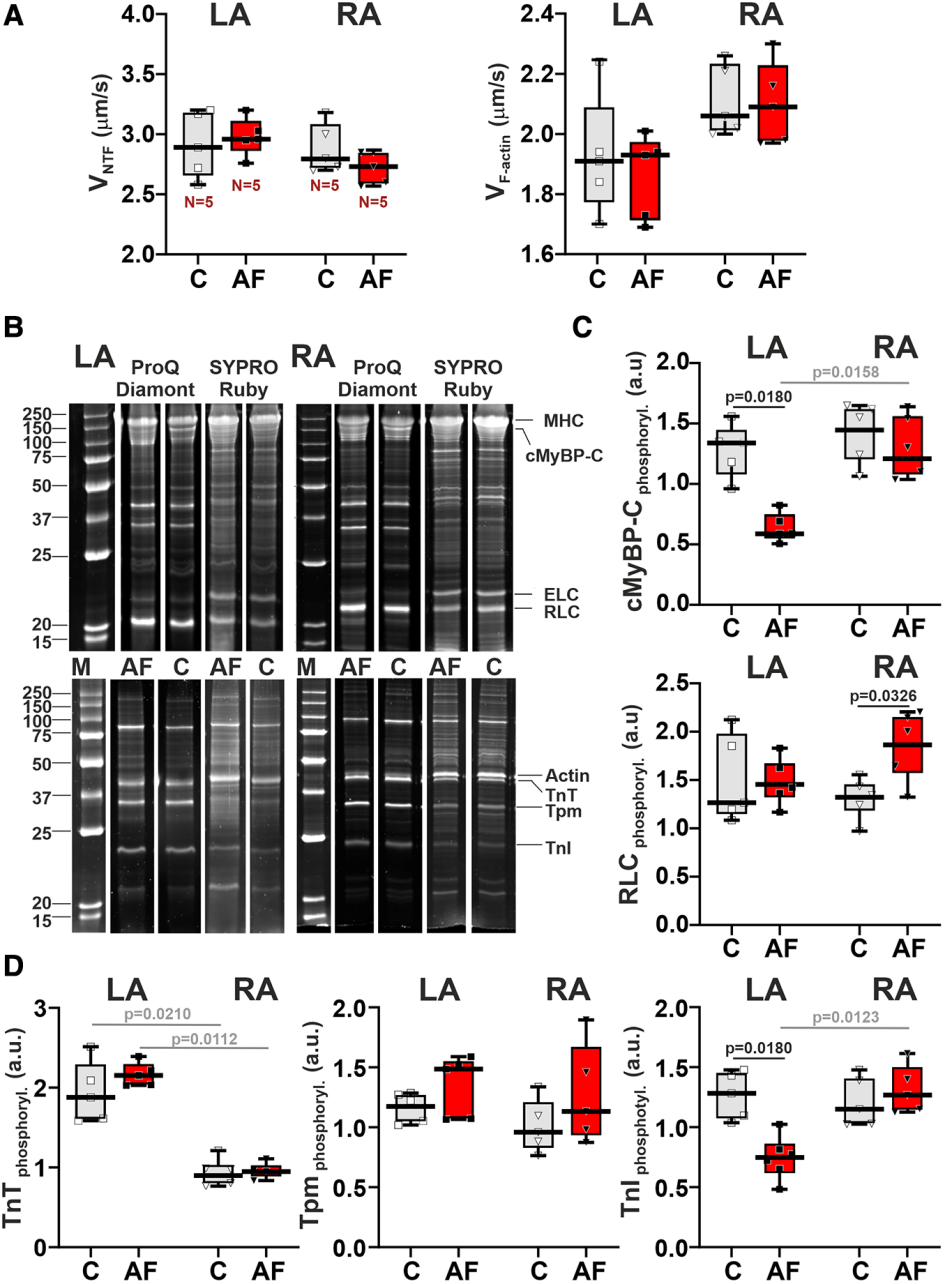
AF-induced changes in actin-myosin interaction and sarcomeric protein phosphorylation in LA and RA. (**A**) Sliding velocity of native thin filaments (NTF) and F-actin over myosin from LA and RA using the *in vitro* motility assay. (**B**) The example of gel electrophoresis of NTF and myosin extracted from the control (**C**) and in ACh-CaCl_2_-induced AF groups. MHC, myosin heavy-chain; cMyBP-C, cardiac myosin binding protein-C; ELC, myosin essential light chain; RLC, myosin regulatory light chain; TnT, troponin T; Tpm, tropomyosin; TnI, troponin I. Phosphorylation was assessed using Pro-Q Diamond and SYPRO Ruby (Invitrogen, Eugene, OR, USA). Precision Plus Protein™ unstained Standards (Bio-Rad, Hercules, CA, USA) was used as molecular weight markers for protein (**M**). (**C**) Phosphorylation levels of cMyBP-C and RLC. (**D**) Phosphorylation levels of TnT, Tpm, and TnI. Data are presented in box and whisker plots, where the boxes are drawn from Q1 to Q3, horizontal lines represent median values and whiskers provide the 100% range of the values. Each dot represents a median value from one animal. The number of *N* hearts in each group is shown below the first boxplot. Scheirer-Ray-Hare test with Bonferroni *post hoc* test.

To reveal molecular mechanisms of AF effects on the contractile function of atrial CM, we examined changes in actin-myosin interaction. We analyzed the sliding velocity of native thin filaments (NTF) and F-actin over myosin from LA and RA in the *in vitro* motility assay. AF did not affect either the maximum velocity of NTF (*v*_max_, the sliding velocity at saturating Ca^2+^ concentration) (*p* > 0.54) or the sliding velocity of F-actin (*p* > 0.88) over myosin from both atria (SRH test, Figure 6A). In both control and AF groups, we found no LA vs. RA differences in NTF (*p *> 0.99) or F-actin velocity (*p *> 0.12, SRH test, Figure 6A).

We also analyzed the AF effects on the phosphorylation levels of myosin regulatory light chain (RLC), cMyBP-C (cardiac myosin binding protein-C), troponin T (TnT), troponin I (TnI), and tropomyosin (Tpm) in LA and RA (Figures 6B–D). In LA, AF decreased the phosphorylation level of cMyBPC (∼2.27-fold) and of TnI (∼1.71-fold*, p* = 0.0180), and did not change the phosphorylation of RLC (*p* > 0.99, SRH test, Figures 6C,D). In RA CM, AF did not affect the phosphorylation of cMyBPC (*p* = 0.6733) and TnI (*p* = 0.7226, Figures 6C,D), while the phosphorylation level of RLC was increased (∼1.39-fold*, p* = 0.0326). TnT and Tpm phosphorylation levels did not differ between the AF and control groups in both LA and RA (*p* > 0.71 for TnT and *p* > 0.24 for Tpm).

In the control and AF groups, we observed the LA vs. RA difference in TnT phosphorylation level (*p* = 0.0210 for the control group, *p* = 0.0112 for AF, Figure 6D). An AF-induced decrease in cMyBPC and TnI phosphorylation provoked inter-atrial differences in their phosphorylation levels with the values being smaller in LA than in RA (*p* = 0.0158 for cMyBPC and *p* = 0.0123 for TnI, Figures 6C,D).

Thus, AF provoked a decrease in the phosphorylation levels of cMyBP-C and TnI in LA and an increase in the phosphorylation level of RLC in RA, inducing inter-atrial differences in post-translational modifications of contractile proteins.

## 4. Discussion

In the heart, LA and RA work under different mechanical and metabolic environments: LA contracts against a higher pressure compared to RA and receives oxygenated blood from the lungs, whereas RA receives deoxygenated blood from the body. These LA vs. RA differences may cause an intrinsic diversity in structure as well as in functional properties between LA and RA CM in norm and provoke their different responsiveness to pathological conditions. In this study, we analyzed the differences in the characteristics of mechanical function between LA and RA CM in paroxysmal AF using an ACh-CaCl_2_-induced AF rat model.

The main findings of our study are as follows: (i) AF provokes morphological changes and sarcomeric dysfunction in LA myocardium, which is associated with the increased [ROS]*_i_*, reduced [NO]*_i_*, and decreased phosphorylation of cMyBP-C and TnI; (ii) AF induces LA-to-RA differences in wall thickness, myofibrillar content, EDSL, and sarcomere shortening-relengthening velocities, which could be related to inter-atrial differences in ROS production and contractile protein function but not to changes in [Ca^2+^]*_i_* transients. (iii) mechanical load may regulate contractile function of LA and RA CM in AF affecting the auxotonic force characteristics in RA CM.

### 4.1. The characteristics of the paroxysmal AF model

The clinical course of AF is progressive. It first occurs as a paroxysmal form with short-lasting AF episodes, which last <7 days and terminate spontaneously, but over time, it becomes chronic with long-lasting persistent AF episodes. Paroxysmal AF differs from persistent forms, including changes in atrial structure and function and the pathophysiological importance of the pulmonary vein sleeves (35, 36). Both paroxysmal and persistent AF may lead to the structural, electrical and mechanical remodeling of atrial myocardium. In this study, we analyzed the mechanical characteristics of LA and RA CM using the 7-day AF rat model induced by ACh-CaCl_2_ injections. The arrhythmogenic phenotype of the used AF model is studied in detail by other authors (22, 37). Autonomic nervous system activation is the risk factor for AF. Studies on patients and animal models showed that AF onset is often associated with combined sympatho-vagal activation (38). The combination of ACh with CaCl_2_ induces AF in animals through the activation of M2 receptors (cholinergic stimulation) and the influence of high Ca^2+^ concentrations (adrenergic stimulation). The actions of ACh are quickly terminated by the activity of cholinesterase, which hydrolyzes ACh. However, the first minutes of M2 receptor activation are sufficient for the induction of immediate early gene expression by activation of protein kinase C (PKC) (39). ACh stimulates M2 receptors that result in the activation of ACh-activated K^+^ current leading to a reduction in AP duration (5). ACh also leads to ROS overproduction (40), which activates redox-regulated signaling enzymes (e.g., PKC and protein kinase A), resulting in changes in protein phosphorylation (41, 42). Alterations in the CM redox state have been closely linked to the initiation, development, and maintenance of AF (43–45).

Elevated extracellular Ca^2+^ concentration may increase SR Ca^2+^ load (46) leading to focal ectopic activity (47). Additionally, high extracellular Ca^2+^ levels may increase the release of norepinephrine from sympathetic end terminals (48, 49). This together results in a decrease in the atrial effective refractory period creating an atrial substrate for AF and in an increase of the incidence of inducible AF and its duration, reflecting the pathological process of AF (8, 21).

Consistently with other studies on patients and animals, we have shown that ROS production in atrial CM is increased in AF (50, 51), while NO production is decreased (11, 52–54). An imbalance in NO production is involved in the pathology of AF (55). NO post-translationally modifies proteins through S-nitrosylation (56) or activates the soluble guanylate cyclase (sGC)/cyclic guanosine monophosphate (cGMP)/protein kinase G (PKG) phosphorylation pathway, which results in protein phosphorylation (57).

Experimental studies on atrial biopsies from patients with AF and animal models revealed that alterations in intracellular Ca^2+^-handling in atrial CM play an important role in AF pathophysiology (14, 47). In contrast to previous studies on animals under rapid atrial pacing (20, 58) and patients with paroxysmal AF (59), we have shown that AF induced by ACh-CaCl_2_ did not provoke significant changes in [Ca^2+^]*_i_* transients in atrial CM. Here we used hierarchical techniques to take into account clustering of data taken from each animal, and consistently with (34) we found that [Ca^2+^]*_i_* transient parameters were highly clustered (>50%). Standard statistical methodologies for independent data points, if used instead, suggested that [Ca^2+^]*_i_* transient amplitudes were reduced in AF in both LA and RA CM. Thus, statistical tests contribute to the data inconsistencies. Moreover, regional heterogeneity within CM also may affect the results obtained. Greiser et al. showed that while [Ca^2+^]*_i_* transient amplitudes in the subsarcolemmal areas of atrial CM were unaltered during short-term AF, the [Ca^2+^]*_i_* transient amplitudes in the center of CM were reduced (58).

The Ca^2+^ release from the SR evoked by a rapid caffeine application were comparable between the control and AF groups, in agreement with (58, 60). Note that in LA CM from the AF group there was a high data variability with individual SR Ca^2+^ load values being much greater than the median value. Thus, our study shows that Ca^2+^ cycling in atrial CM was unaltered in paroxysmal AF. Further work is needed to evaluate Ca^2+^ cycling in detail, e.g., calcium sparks, sodium-calcium exchanger (NCX) or SERCA2a activity. It has been shown that *β*-adrenergic stimulation may affect differently cAMP-dependent PKA signaling in patients in sinus rhythm and in patients with AF that might regulate the phosphorylation of specific Ca^2+^-handling proteins (61).

In contrast to unchanged [Ca^2+^]*_i_* transients, we found that ACh-CaCl_2_ induced AF impaired the contractility of single atrial CM consistently with data obtained in dogs under rapid atrial pacing (20, 62). Leistad et al. at the whole swine heart showed that contractility of LA was increased during the first seconds after AF up to 15 min, while a subsequent phase of reduced atrial contractility occurs if the AF is sustained more than 5 min (63). Thus, impaired CM contractility, which probably is inherent to the progression from the paroxysmal AF to sustained AF (62) contributes to prothrombotic atrial hypocontractility in AF (47, 64).

Changes in atrial structure can increase the likelihood of both ectopic activity and re-entry through abnormal electrical conduction (35). Consistently with other studies on short-term AF, we found no significant changes in CM width (65, 66). However, CM length was longer in LA in the AF group pointing to the development of atrial dilatation in ACh-CaCl_2_ induced AF model. AF resulted in a reduced myofibrillar content and an increased glycogen content in atrial CM, which is in agreement with data obtained on patients with permanent AF (2). We also measured collagen content to examine atrial fibrosis after 7-day AF. Our results showed fibrosis in LA that was consistent with the previous results obtained in ACh-CaCl_2_ induced AF in mice (66), rapid atrial pacing in dogs (67) and in patients with paroxysmal AF (68, 69). Note, that the relationship between the course of AF and atrial fibrosis is complex and nonlinear, so the AF paroxysm frequency and its progression toward the persistent or permanent form are not always associated with the atrial fibrosis degree (70).

### 4.2. LA vs. RA differences in CM mechanical function in short-term AF

In the control rats, we found no significant LA vs. RA differences either in the characteristics of [Ca^2+^]*_i_* transients, SL dynamics in mechanically non-loaded CM, or in parameters of auxotonic force in mechanically loaded CM, while CM length was shorter in LA than in RA. In the *in vitro* motility assay, the velocity of F-actin and native thin filaments over myosin did not differ between the control LA and RA groups according to the non-different velocity of sarcomere shortening observed in single CM.

We have demonstrated here that AF provoked inter-chamber differences in the structure and function of LA and RA. Our results are in agreement with the conception that LA may play an important role in the development and maintenance of AF (9, 10, 71). In LA, AF resulted in morphological changes with the CM elongation, increased collagen and glycogen deposition, and a decreased myofibrillar content. In mechanically non-loaded LA CM, AF provoked a decrease in the sarcomere shortening amplitude and a reduction in the velocities of sarcomere relengthening. In RA, AF also led to a decrease myofibrillar content and an increase in glycogen deposition but did not change the sarcomere shortening amplitude increasing the velocity of sarcomere shortening in single CM. These changes resulted that in AF, LA had a thicker atrial wall, a smaller myofibrillar content, shorter end-diastolic SL, and slower sarcomere shortening and relengthening compared to RA.

We showed that AF provoked a decrease in the phosphorylation of total cMyBP-C and TnI in LA leading to LA vs. RA differences in cMyBP-C and TnI phosphorylation during AF. Decreased phosphorylation of cMyBP-C was shown to reduce the amplitude as well as the velocity of mechanically non-loaded CM contraction and relaxation in mice (72). Decreased cTnI phosphorylation also contributes to impaired systolic function as well as myocardial relaxation (73, 74). Thus, our results suggest that decreased phosphorylation of cMyBP-C and TnI contributes to depressed sarcomere shortening, along with a reduced velocity of sarcomere relengthening obtained in LA CM, and may underlie inter-atrial differences in the characteristics of SL dynamics during AF. No changes were found in cMyBP-C and TnI phosphorylation levels in RA, consistently with data on goats with AF induced by rapid atrial pacing for 10 days (75). The enhanced RLC phosphorylation was shown to increase the amplitude and velocity of CM shortening (76). Probably, an AF-induced increase in RLC phosphorylation in RA contributes to increased velocity of sarcomere shortening and may be protective for preserved sarcomere shortening in RA CM during AF.

ROS production regulates mechanical function of atrial CM (77), contributing to the development of contractile dysfunction (78). We found that AF provoked the LA vs. RA difference in [ROS]*_i_* with a greater extent in LA CM than in RA CM. We suggest that the inter-atrial difference in the AF-induced ROS production may contribute to the LA v*s.* RA difference in the phosphorylation of contractile proteins resulting in the different vulnerability of LA and RA CM to AF.

The heterogeneous effects of ACh on atrial CM ion channels also might contribute to observed inter-atrial differences. For instance, in the rat heart, LA has a higher mRNA level of M_2_ receptor than RA (79) that may result in a larger I_KACh_ density providing greater sensitivity to ACh (5, 80). In contrast to current findings, our previous study have shown that 10–15 min incubation of single CM with ACh decreased the time to peak sarcomere shortening, time to 50% relengthening mainly in LA CM than in RA CM without significant effects on the sarcomere shortening and [Ca^2+^]*_i_* transient amplitudes (24). Probably, ACh-CaCl_2_-induced AF associated with the combined sympatho-vagal activation for 7 days provokes changes in the myocardial structure mainly in LA leading to the different responsiveness of LA and RA CM and differences in the results obtained.

Atrial contractile force is influenced by atrial preload and atrial afterload. Increased mechanical load also is an important trigger for atrial remodeling (81, 82). AF is frequently associated with atrial elongation caused by pressure or volume overload (82, 83). In contrast to our observations on mechanically non-loaded CM, we showed that short-term AF resulted in the depressed auxotonic force amplitude and kinetics in RA CM, while force characteristics were preserved in LA CM. The inter-atrial differences in the stress response between LA and RA CM, which were recently demonstrated (84) may contribute to observed results and warrant further studies.

## 5. Conclusions

We have shown that inter-atrial differences are inherent characteristics of atrial myocardium in AF. Mechanically non-loaded LA CM are more vulnerable to paroxysmal AF than RA CM that could be attributed to a greater increase in ROS production, a decrease in myofibrillar content, and changes in sarcomeric protein phosphorylation in LA. However, mechanical load changes the contractile responses of LA and RA CM in AF. The observed differences could be related to the chamber-specific sarcomeric dysfunction rather than to changes in Ca^2+^ handling. We suggest that the appearance of LA vs. RA differences in morphological and mechanical characteristics after 7 days of paroxysms may contribute to a progression from paroxysmal to sustained forms of AF.

### 5.1. Limitation

This study has the following limitations. The AF model we applied does not explain the overall cause of paroxysmal AF. Ectopic activity arising from the pulmonary veins plays a particularly important role in paroxysmal AF in patients, while its role might be not so pronounced in animal AF models (47). Nevertheless, animal models are useful for testing specific hypotheses about basic mechanisms and uncovering mechanistic components for further testing in human studies. Our study is limited to male rats. There may be a sex difference in the myocardial remodeling (85) affecting LA vs. RA differences. Experiments on mechanically loaded LA and RA CM were performed with the same stiffness of carbon fibers, although LA and RA CM are subjected to the different mechanical load *in vivo*. Further research will be devoted to the effects of various mechanical loads on the contractile function of atria in AF. In addition, an analysis of Ca^2+^-regulating proteins, such as ryanodine receptor, NCX, SERCA2a, and calmodulin is further needed to conciliate data inconsistencies.

## Data Availability

The raw data supporting the conclusions of this article will be made available by the authors, without undue reservation.
