# Supplementary material for: The inter-chamber differences in the contractile function between left and right atrial cardiomyocytes in atrial fibrillation in rats
Source: Front Cardiovasc Med. 2023 Aug 7;10:1203093. doi: 10.3389/fcvm.2023.1203093 (PMC10440706; doi:10.3389/fcvm.2023.1203093)
Supplement: Supplementary file 1 [file Datasheet1.doc]

|  |
| --- |
| 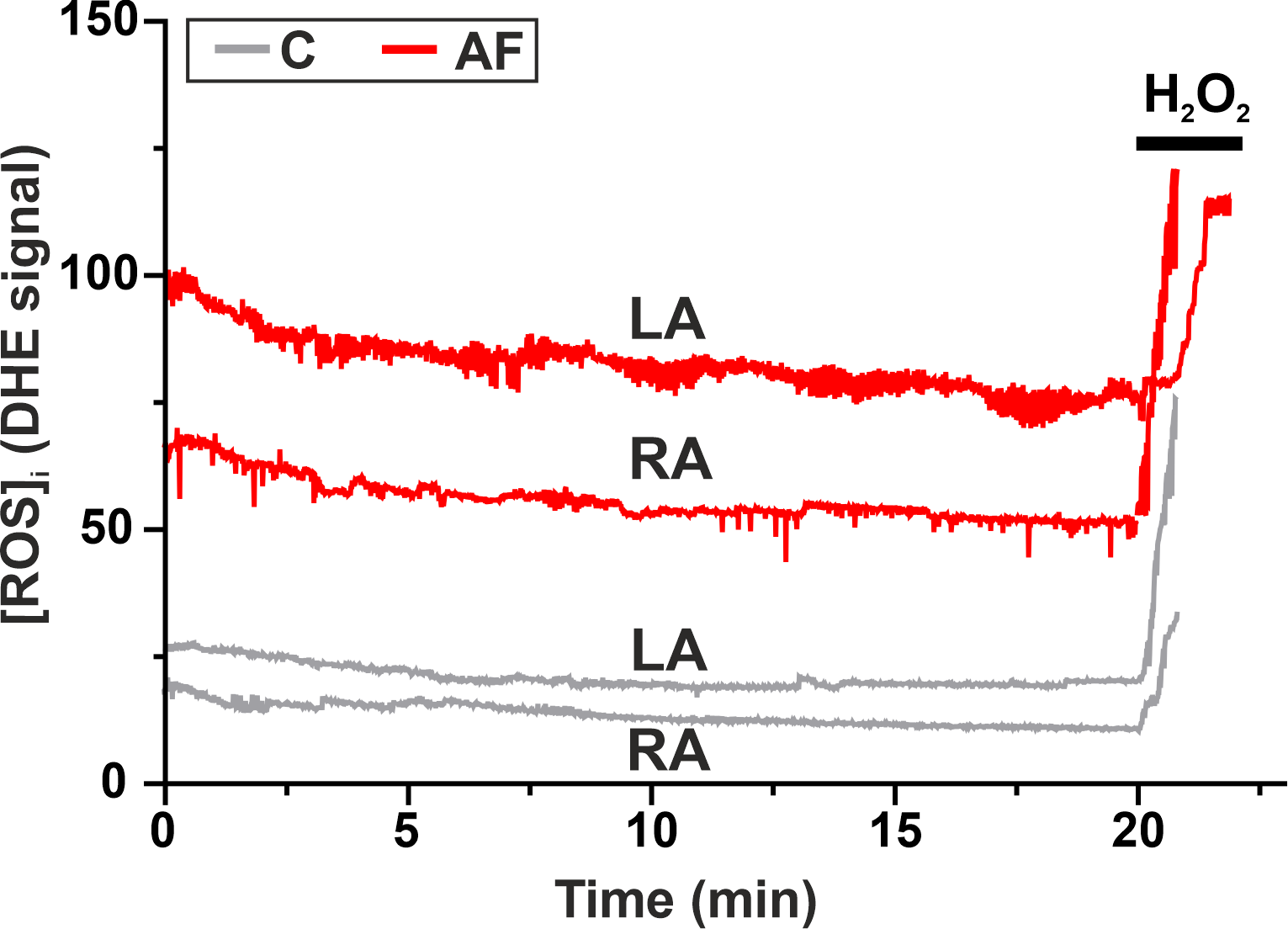  **Figure S1.** Сhanges of DHE signal in electrically stimulated (at 1 Hz) single cardiomyocytes (CM) from the left (LA) and right atria (RA) in control rats (C) and rats with ACh-CaCl_2_-induced AF. H_2_O_2_ (1 mM) was applied to increase ROS production. The intensity of emitted fluorescence was collected from a selected narrow region on the CM surface (3 pixels high, 200 pixels length).  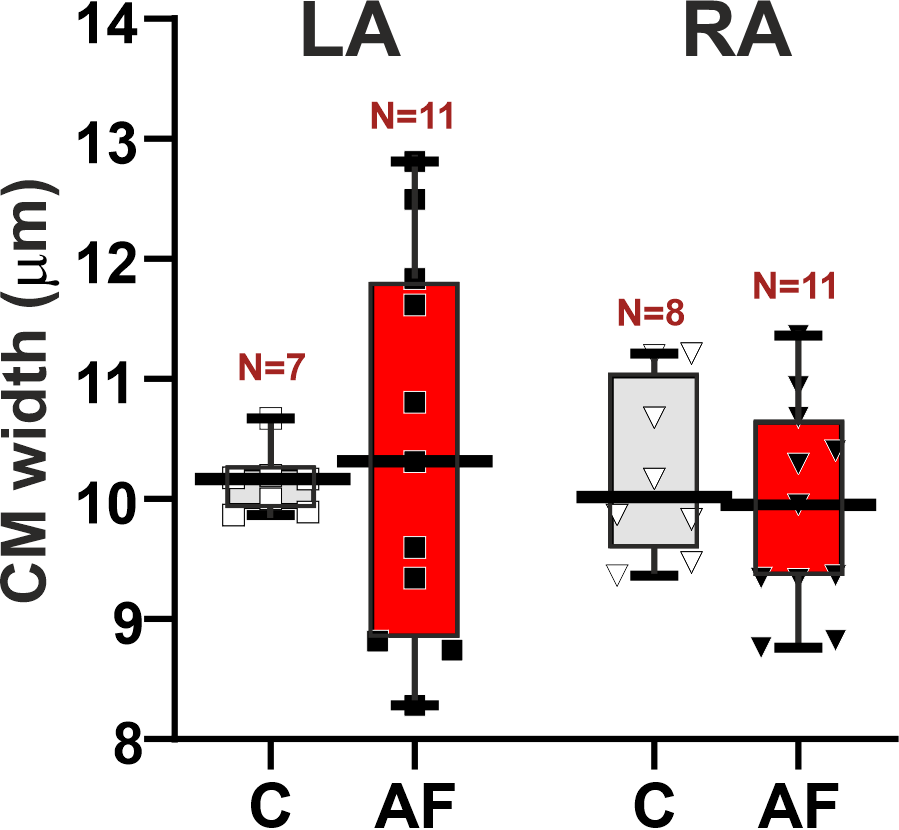  **Figure S2.** Width (diameter) of single cardiomyocytes (CM) from the left (LA) and right atria (RA) in control rats (C) and rats with ACh-CaCl_2_-induced AF. Data are presented in box and whisker plots, where the boxes are drawn from Q1 to Q3, horizontal lines represent median values and whiskers provide the 100% range of the values. Each dot represents a median value from one animal. The number of N hearts in each group is shown below the boxplot. Scheirer-Ray-Hare test with Bonferroni post-hoc test. |


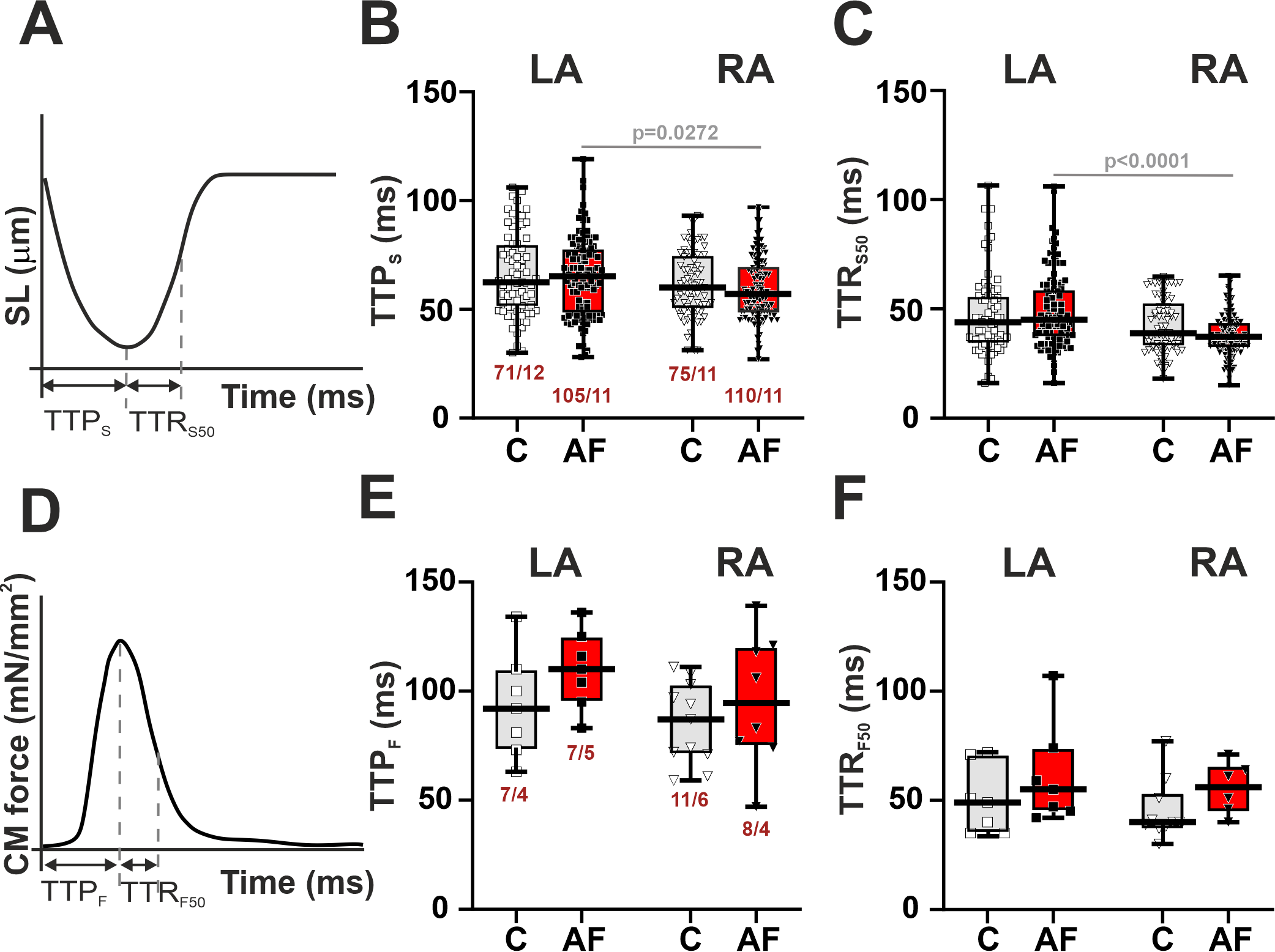


**Figure S3**. The effects of ACh-CaCl_2_-induced AF on the characteristics of auxotonic force (in mechanically loaded CM) and sarcomere length (SL) dynamics (in mechanically non-loaded CM) in LA and RA CM. (A) Analyzed parameters derived from the SL change signal. (B) Time to peak shortening (TTP_S_). (C) Time from peak shortening to 50% sarcomere relengthening (TTR_S50_). (D) Analyzed parameters derived from the auxotonic force signal. (E) Time to peak force development (TTP_F_). (F) Time from force peak to 50% relaxation (TTR_F50_). Data are presented in box and whisker plots, where the boxes are drawn from Q1 to Q3, horizontal lines represent median values and whiskers provide the 100% range of the values. Each dot represents an individual CM. The number of n CM from N hearts in each group is shown. Statistical significance was determined by hierarchical clustering analysis with log or square root-transformed data.


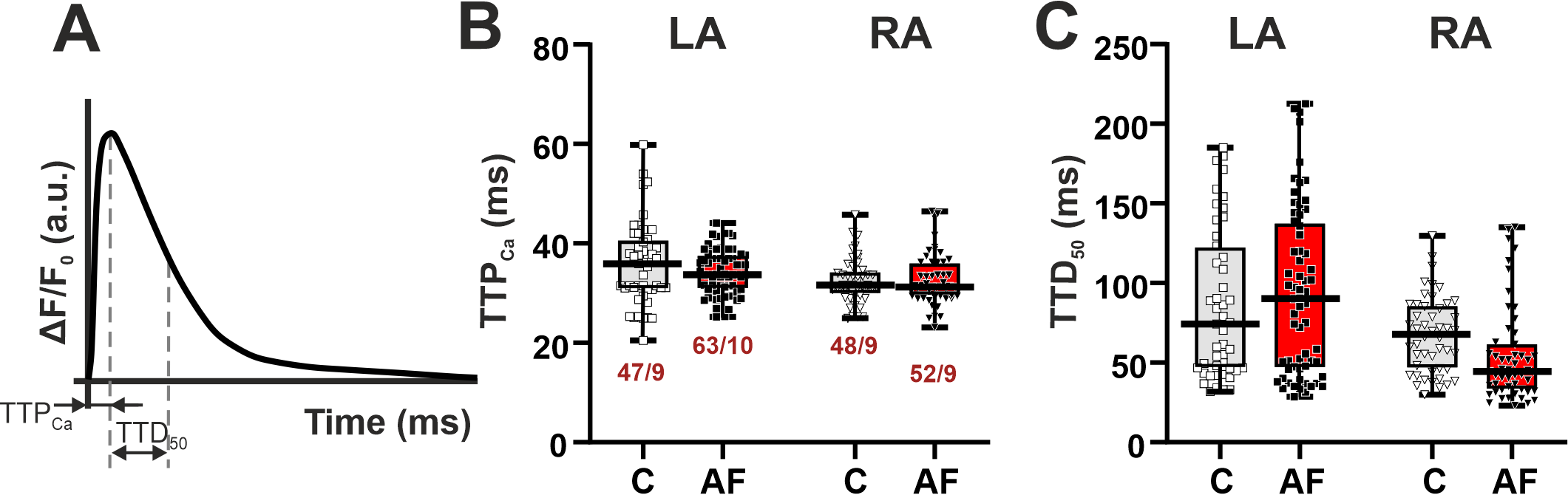


**Figure S4**. No LA *vs.* RA differences in [Ca^2+^]_i_ transients in rats with ACh-CaCl_2_-induced AF. (A) Analyzed parameters derived from the [Ca^2+^]_i_ change (F/F0) signal. (B) Time to peak [Ca^2+^]_i_ transients (TTP_Ca_) in contracting LA and RA CM from the control rats (C) and AF rats (AF). (C) Time to 50% decay of [Ca^2+^]_i_ transients. Data are presented in box and whisker plots, where the boxes are drawn from Q1 to Q3, horizontal lines represent median values and whiskers provide the 100% range of the values. Each dot represents an individual CM. The number of n CM from N hearts in each group is shown. Hierarchical clustering analysis with log-transformed data.
